# Hesperidin Attenuates Deltamethrin-Induced Testicular Damage by Modulating Apoptosis-Associated Signaling and Profibrotic Gene Expression: Biochemical, Molecular, and Histological Evidence

**DOI:** 10.3390/life16081363

**Published:** 2026-08-19

**Authors:** Halime Tuba Canbaz, Burcu Gultekin, Ilknur Cinar Ayan, Hasan Basri Savas, Furkan Adem Canbaz, Gokhan Cuce, Serpil Kalkan

**Affiliations:** 1 Department of Histology and Embryology, Hamidiye Faculty of Medicine, University of Health Sciences, 34668 İstanbul, Türkiye; 2 Department of Histology and Embryology, Faculty of Medicine, Necmettin Erbakan University, 42090 Konya, Türkiye; bgultekin@erbakan.edu.tr (B.G.); gcuce@erbakan.edu.tr (G.C.); skalkan@erbakan.edu.tr (S.K.); 3 Department of Medical Biology, Faculty of Medicine, Necmettin Erbakan University, 42090 Konya, Türkiye; icinar@erbakan.edu.tr; 4 Department of Medical Biochemistry, Faculty of Medicine, Mardin Artuklu University, 47200 Mardin, Türkiye; hasansavas@artuklu.edu.tr; 5 Department of Pediatric Urology, Sancaktepe Sehit Prof. Dr. Ilhan Varank Training and Research Hospital, 34785 İstanbul, Türkiye; furkanadem.canbaz@saglik.gov.tr

**Keywords:** deltamethrin, hesperidin, testicular toxicity, histopathology, oxidative stress, apoptosis

## Abstract

This study aimed to investigate the effect of hesperidin (HSP) at two different doses on deltamethrin (DLM)-induced testicular damage. The study comprised four groups of Wistar Albino rats (*n* = 8 per group): Control, DLM, DLM + HSP 100 mg/kg, and DLM + HSP 300 mg/kg. At the end of the study, oxidative stress markers and CRP were measured in serum samples. Testicular tissues were assessed histopathologically. Expression of Bcl-2, Bax, and Nrf-2 were determined by immunofluorescence staining. RT-qPCR assessed tissue collagen (*COL1A1*, *COL3A1*) and apoptotic () expressions. HSP at both doses lowered the DLM-induced increase in total oxidant status, oxidative stress index (total oxidant status/total antioxidant capacity), and CRP levels. Nrf-2 was increased by a high dose of HSP (300 mg/kg). Whereas HSP normalized other apoptotic parameters at both doses, it partially normalized Bax expression in immunofluorescence and Bcl-2 expression in RT-qPCR only at 300 mg/kg. HSP reduced the increased fibrosis in DLM exposure, as revealed by the expressions of *COL1A1* and *COL3A1* at both doses. HSP partially ameliorated DLM-associated histological alterations, including Johnsen’s score, tubule diameter, and thickness of the tunica albuginea. HSP may exert protective effects against DLM-induced testicular injury by modulating systemic oxidative status, apoptosis-associated signaling, and profibrotic gene expression.

## 1. Introduction

Pyrethroids are synthetic insecticides employed for many applications. They are extensively utilized for the management of agricultural vectors and the control of household pests. Deltamethrin (DLM) with a chemical formula of C_22_H_19_Br_2_NO_3_ is a potent Type II pyrethroid within this classification [1]. DLM can easily pass through biological membranes in organisms due to its high lipophilic structure, and it functions effectively as an insecticide. Frequent consumption and environmental exposure provide a substantial public health issue and are growing more prevalent [2]. DLM has neurotoxic, hepatotoxic, nephrotoxic, endocrine-disrupting, and oxidative stress-inducing toxic effects [3]. Increased reactive oxygen species (ROS) and reactive nitrogen species, stemming from oxidative stress induced by DLM exposure, disturb the oxidant–antioxidant equilibrium, leading to cellular damage, cell death, and apoptosis [4]. The Nuclear factor erythroid 2–related factor 2 (Nrf-2) transcription factor pathway has been recognized as essential to this mechanism of action. DLM is reported to affect the oxidative state via altering Nrf-2 expression. Moreover, exposure to DLM may result in persistent inflammation and fibrosis. Oxidative stress and the endocrine imbalance associated with all these mechanisms can impair spermatogenesis by affecting the hypothalamus–pituitary–gonadal axis and cause histopathological damage in testicular tissue [5,6]. A recent study demonstrated that the short-term consumption of DLM-contaminated fruit can also cause testicular toxicity even at low concentration [7]. In this context, the potential for testicular toxicity of DLM is reported to pose a serious risk to male reproductive health [8]. Some natural products, such as oat oil and Ginkgo biloba, were studied in the literature against DLM-induced toxicity [9,10].

Hesperidin (HSP) (3,5,7-trihydroxy flavanone-7-rhamnoglucoside) is a flavonoid commonly included in citrus fruits. Its therapeutic function has attracted attention owing to its antioxidant, anti-inflammatory, and anticancer attributes, in addition to its capacity to impede many forms of pathogenesis [11]. It has been extensively investigated for its potential therapeutic effects in the treatment of type 2 diabetes mellitus, cardiovascular diseases, cancer, and neurological and psychiatric disorders [12]. The protective effects of HSP have been reported in different experimental models of testicular injury, such as diabetes or cyclophosphamide-induced testicular damage [13,14]. However, its protective efficacy in DLM-induced testicular injury, particularly in a dose-dependent manner, remains largely unexplored. Furthermore, the interplay among systemic oxidative stress markers, apoptosis-associated signaling, profibrotic molecular changes, and histopathological alterations in DLM-induced testicular injury has not been comprehensively investigated.

In this study, the potential efficacy of HSP administration at different doses against DLM-related testicular toxicity, with respect to systemic oxidative status, apoptosis-associated signaling, and profibrotic gene expression, were investigated. The alterations induced by the previously stated pathways were assessed using biochemical analysis and RT-qPCR, and these changes were corroborated at the tissue level via histological evaluation and immunofluorescence assessment. We hypothesized that HSP would reduce testicular damage via these mechanisms.

## 2. Materials and Methods

### 2.1. Animals and Experimental Design

The experiment comprised 32 male Wistar Albino rats. Four groups were randomly formed, each including eight rats. Their age was four months, and their weights varied from 250 to 300 g. The animals were provided with unrestricted access to tap water and standard laboratory food. The rats were housed in an environment maintained at 24 ± 1 °C, with a humidity level of 45 ± 5%, and subjected to a 12 h light/dark cycle.

Group 1 (Control): A total of 1 mL of corn oil was included in the normal diet daily during the study; no interventions were performed.
Group 2 (DLM): DLM was administered daily during the study.
Group 3 (DLM + HSP 100 mg/kg): DLM and HSP at a dose of 100 mg/kg were administered daily during the study.
Group 4 (DLM + HSP 300 mg/kg): DLM and HSP at a dose of 300 mg/kg were administered daily during the study.

All of corn oil, DLM, and HSP were applied via oral gavage. The duration of the study was 30 days. The dose of DLM (1.28 mg/kg) was determined according to the literature [15]. Two different doses of HSP (100 mg/kg, 300 mg/kg) were also selected based on the literature to assess dose-dependent efficacy [16]. DLM (45423 Supelco, CAS No: 52918-63-5) and HSP (H5254 Sigma Aldrich, CAS No: 520-26-3, assay ≥80% by HPLC) were freshly prepared daily prior to administration. Due to its low aqueous solubility, HSP was prepared as a uniform suspension in distilled water at two different concentrations of 10 mg/mL and 30 mg/mL (administered volume: 10 mL/kg). To maintain homogeneity during dosing, the suspension was subjected to sonication/vortexing for 15 min and kept under continuous magnetic stirring immediately prior to administration. DLM was freshly prepared by dissolving it in corn oil and was first administered once daily by oral gavage at a dose of 1.28 mg/kg. The dose was adjusted daily according to the body weight of each animal throughout the experimental period. HSP, solubilized in distilled water, was administered 30 min thereafter.

General anesthesia was created at the end of the study. Ketamine HCl (50 mg/kg) and Xylazine HCl (10 mg/kg) were given intraperitoneally for obtaining anesthesia. Under anesthesia, blood samples were collected with cardiac puncture, and all rats were then sacrificed via the cervical dislocation method. The testicular tissues were examined macroscopically for gross abnormalities (discoloration, edema, hemorrhage, focal lesions, or marked atrophy) prior to tissue collection and removed. No overt macroscopic abnormalities were noted. Following tissue collection, one testis from each animal was allocated for histopathological and immunofluorescence analyses, whereas the contralateral testis was used for RNA extraction and gene expression analysis. All subsequent evaluations were performed in a blinded manner.

### 2.2. Biochemical Analysis

The blood samples were transferred into gel separator biochemistry tubes. The samples were centrifuged at 1500 *g* for 10 min, resulting in the acquisition of sera. Thereafter, the sera were aliquoted and preserved at −80 °C in Eppendorf tubes until analysis. Serum samples were dispatched to the biochemistry laboratory unmarked with group identities and evaluated blindly by the investigator. Before biochemical testing, all serum samples were concurrently thawed and equilibrated at room temperature. Each sample was gently vortexed to provide homogeneity. The serum of each animal was used as a separate biological replicate. Due to the limited amount of serum obtained from each animal and ethical considerations aimed at minimizing animal use, additional biological replicates were not possible within the current experimental design. The analyses were performed according to the manufacturer’s instructions, and samples were reanalyzed when assay quality criteria were not met.

The total antioxidant capacity (TAC) and total oxidant status (TOS) were assessed by fully automated colorimetric techniques (Rel Assay Diagnostics, Gaziantep, Turkey) [17,18]. The TAC levels were quantified in micromolar Trolox equivalents (mmol Trolox eq/L). The TOS levels were documented as micromolar hydrogen peroxide equivalent (µmol H_2_O_2_ eq/L). The oxidative stress index (OSI) was subsequently calculated using the formula: OSI = TOS/TAC. The blood C-reactive protein (CRP) was measured with the Abbott Architect c8000 analyzer and kits from Abbott Laboratories, North Chicago, IL, USA. CRP values were documented in mg/L.

### 2.3. RNA Extraction and Real-Time Quantitative PCR (RT-qPCR) Analysis

In the RT-qPCR assay, all equipment utilized was sterilized with 70% alcohol prior to dissection to reduce RNA degradation. Low-temperature resistant, nuclease-free cryovial tubes (2 mL) required for tissue storage were labeled prior to dissection, and the experiment commenced following the preparation of liquid nitrogen. Following euthanasia, testicular tissues were promptly excised and segregated from blood and adipose tissues (maximum 1–2 min). Following a brief rinse with RNase-free PBS, the tissues were sectioned and promptly transferred into cryovial tubes. The tubes were subsequently positioned in the liquid nitrogen tank with haste. Testicular tissues were preserved at −80 °C until the RNA extraction phase. Total RNA was extracted from testicular tissues for the investigation of Bcl-2-associated X protein (*Bax*), B-cell lymphoma 2 (*Bcl-2*), *COL1A1*, and *COL3A1* gene expression (GeneAll, RiboEX, 301–001, GeneAll, Seoul, Republic of Korea). Each tissue RNA sample underwent treatment with DNase I enzyme to eliminate potential DNA contamination (Thermo Fisher Scientific, #EN0521, Waltham, MA, USA). cDNA synthesis was conducted following the manufacturer’s guidelines (BIO-RAD, 170–8891, Hercules, CA, USA). Primer sequences for the chosen target and housekeeping genes were created via the IDT PrimerQuest online tool (https://www.idtdna.com/PrimerQuest/Home/Index, accessed on 19 August 2026). The *GAPDH* gene served as the housekeeping gene for normalization purposes (Table 1). RT-qPCR studies were performed with 5×HOT FIREPol^®^ EvaGreen^®^ qPCR Master Mix Plus (ROX) (Solis BioDyne, Tartu, Estonia) on a Bio-Rad CFX Connect™ Real-Time System. The PCR amplification cycle was conducted under the specified conditions: Following an initial activation phase of 12 min at 95 °C, 40 cycles are conducted consisting of 15 s at 95 °C, 20 s at 60 °C, and 20 s at 72 °C.

### 2.4. Histopathological Analysis

Histopathological evaluation was performed using testicular tissues obtained from all animals in each experimental group (*n* = 8 per group). The testicular tissues were evaluated macroscopically and preserved in 10% formalin. Following tissue preparation, the tissues were embedded into paraffin, and 5 μm-thick sections were obtained from the paraffin blocks. Histopathological analysis used Masson’s trichrome staining to evaluate fibrosis, while hematoxylin-eosin (H&E) staining was employed to determine the Johnsen score, diameter of the seminiferous tubule, and thickness of the tunica albuginea. A histopathological examination was conducted using a double-blind approach using a Zeiss Lab.A1 light microscope (Zeiss, Oberkochen, Germany).

Sections underwent xylene treatment (three times for 20 min each) followed by a declining series of alcohol concentrations (100%, 96%, and 70%) for deparaffinization. The specimens were stained with Harris hematoxylin (Bio Optica 05-06004, Milano, Italy) for 4 min and counterstained with 1% aqueous eosin (Bio Optica 05-10007, Milano, Italy) for 2 min. H&E staining was utilized to evaluate the overall histological structure and determine spermatogenesis. The Johnsen testicular scoring system was utilized for the quantitative evaluation of spermatogenesis [19]. This grading method assesses each seminiferous tubule on a scale from 1 (complete loss of germ cells) to 10 (normal spermatogenesis), based on the arrangement of germinal epithelial cells. Average scores were calculated by examining at least 20 randomly chosen seminiferous tubules from each specimen. Additionally, the diameters of seminiferous tubules and the thicknesses of the tunica albuginea were measured across ten cross-sections of each specimen.

Masson’s trichrome staining was employed to evaluate the increase in connective tissue and fibrotic changes in testicular tissues using the ChemBio (Istanbul, Turkey) staining kit CB6595.0100 [20].

### 2.5. Immunofluorescence Analysis

The sections were affixed on lysine slides. Subsequent to deparaffinization and rehydration, antigen retrieval and endogenous peroxidase inhibition were implemented. Super Block (ScyTek Laboratories, Logan, UT, USA) was also employed to mitigate undesirable nonspecific antigen binding. The sections were treated overnight with Bcl-2 (1:500; mouse monoclonal antibody, sc-7382, Santa Cruz Biotechnology, Dallas, TX, USA), Bax (1:100; mouse monoclonal antibody, sc-23959, Santa Cruz Biotechnology, Dallas, TX, USA), and Anti-NRF2 Rabbit polyclonal antibody (1/1000; GB113808, Servicebio, Wuhan, China) primary antibodies. On the next day, secondary antibodies, Goat Anti-Mouse IgG H&L (Alexa Fluor^®^ 488, ab150113, Abcam, Cambridge, UK) for Bax and Bcl-2, and Goat Anti-Rabbit IgG H&L (1/500; Alexa Fluor^®^ 488, ab150077, Abcam, Cambridge, UK) for Nrf-2 were applied for 1 h at room temperature. Subsequently, slides coated with a mixture comprising PBS, glycerin, and Hoechst 33342 (Thermo Fisher Scientific, Waltham, MA, USA) were examined using a Zeiss Axio microscope (Axiocam 212 color camera, Zeiss, Oberkochen, Germany) equipped with a fluorescent attachment, employing Kameram software version 3.3.0.0 (Istanbul, Turkey) for Bax and Bcl-2 and Zeiss software ZEN 3.12 (Zen lite) for Nrf-2. Ten randomly chosen distinct fields from six sections per animal were evaluated using ImageJ software version 1.54g (National Institutes of Health, Bethesda, MD, USA), yielding an individual mean value for each animal. The average data per animal was employed for statistical analysis, with each animal considered as the experimental unit (*n* = 8 per group). Mean intensity was evaluated following background removal and normalization to tissue/DAPI area.

### 2.6. Statistical Analysis

Statistical evaluation was done using GraphPad Prism (version 8.4.2). Continuous variables were presented as the mean ± SDs. The normality of the data from the groups was evaluated using the Shapiro–Wilk test, histogram, and Q-Q plots. Non-normally distributed data were analyzed using the Kruskal–Wallis test, followed by post hoc Dunn’s test for multiple comparisons. The Brown–Forsythe test was performed to determine the assumption of homogeneity of variances in data that show normal distribution. Then, one-way ANOVA and post hoc Tukey’s test were performed when assumptions of homogeneity of variances were met; otherwise, Welch’s ANOVA and post hoc Dunnet’s T3 test were applied. The 2–ΔΔCT method was used to analyze the relative changes in gene expressions. The significance value of *p* < 0.05 was accepted.

## 3. Results

### 3.1. Biochemical Analyses

Increased TOS levels with DLM were mitigated with HSP treatment at both 100 mg/kg and 300 mg/kg dosages. The mean TOS level was found to be higher in the DLM group (76.9 ± 11.8 μmol/L) compared to the control group (7.1 ± 1.8 μmol/L) (*p* < 0.001). Administration of HSP at both doses (100 mg/kg and 300 mg/kg) decreased TOS levels compared to the DLM group (*p* < 0.001 for both doses). The mean TOS levels in DLM + HSP 100 mg/kg and DLM + HSP 300 mg/kg were 16.8 ± 2.3 μmol/L and 10.5 ± 2.4 μmol/L, respectively. While the mean TOS level of DLM + HSP 100 mg/kg was higher than that of the control group, the mean TOS level of DLM + HSP 300 mg/kg was similar to that of the control group (*p* < 0.001 and *p* > 0.05, respectively).

HSP, at either of the two different doses, did not restore the decrease in TAC induced by DLM. The DLM group exhibited a lower mean TAC level of 1.61 ± 0.21 mmol/L compared to the control group (*p* < 0.001). The mean TAC levels of DLM + HSP 100 mg/kg (1.82 ± 0.27 mmol/L) and DLM + HSP 300 mg/kg (1.91 ± 0.23 mmol/L) were found to be similar to those of the DLM group (*p* > 0.05 for both).

While HSP reduced the OSI levels elevated by DLM at both dosages, this effect was more pronounced at the 300 mg/kg dosage. DLM administration caused an increase in OSI levels compared to the control group with values of 48.23 ± 7.50 and 3.99 ± 0.87, respectively (*p* < 0.001). HSP application at both doses of 100 mg/kg and 300 mg/kg demonstrated a decrease in OSI levels that had increased following DLM (*p* < 0.001 for both). While the mean OSI level of DLM + HSP 100 mg/kg (9.49 ± 2.30) was higher than that of the control group, the mean OSI level of DLM + HSP 300 mg/kg (5.55 ± 1.48) was similar to that of the control group (*p* < 0.001 and *p* > 0.05, respectively).

**Figure 1:**
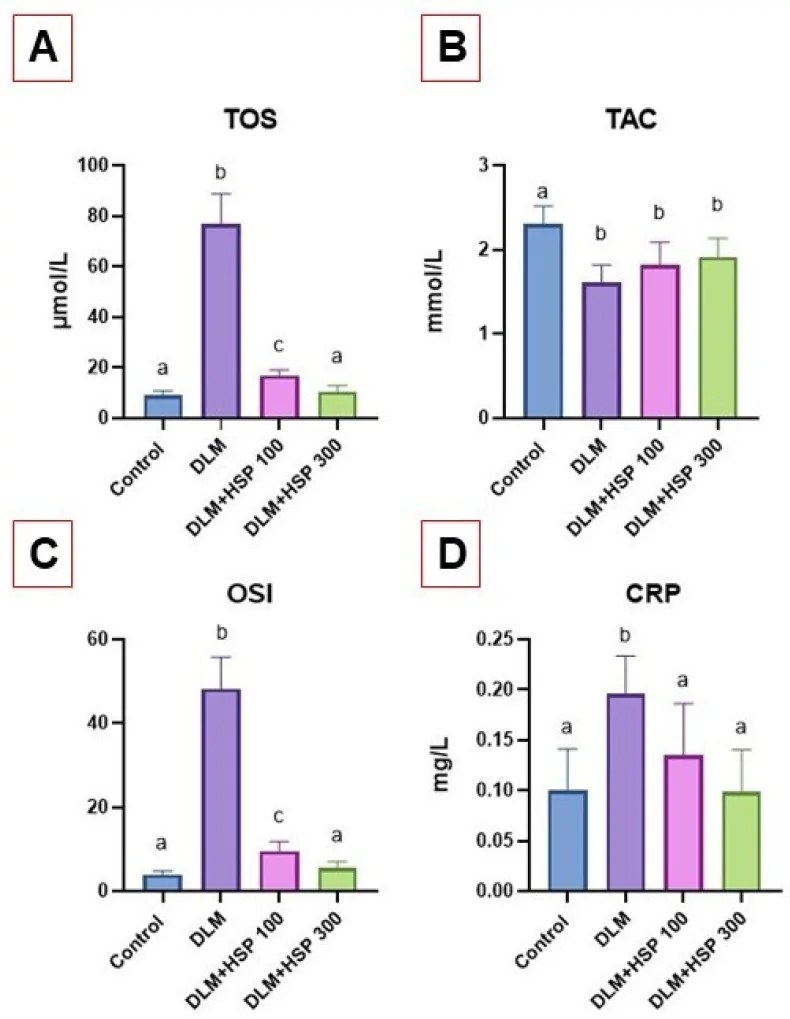
Comparison of biochemical parameters among groups (*n* = 8 animals per group). (**A**) TOS; (**B**) TAC; (**C**) OSI (OSI = TOS/TAC); and (**D**) CRP levels of groups. There is statistically significant difference between groups not sharing the same letter (*p* < 0.05). Data is presented as mean ± SD. Abbreviations: CRP: C-reactive protein; DLM: Deltamethrin; HSP: Hesperidin; OSI: Oxidative stress index; SD: Standard deviation; TAC: Total antioxidant capacity; and TOS: Total oxidant status.

Elevated CRP levels associated with DLM were alleviated by HSP therapy at both 100 mg/kg and 300 mg/kg doses. The mean serum CRP level of the DLM group (0.196 ± 0.037 mg/L) was found to be higher compared to the control (0.1 ± 0.041 mg/L) (*p* < 0.001). HSP administration decreased CRP levels compared to the DLM group (*p* = 0.040 for DLM + HSP 100 mg/kg and *p* < 0.001 for DLM + HSP 300 mg/kg). The mean CRP levels in DLM + HSP 100 mg/kg and DLM + HSP 300 mg/kg were 0.135 ± 0.052 mg/L and 0.099 ± 0.042 mg/L, respectively. Figure 1 depicts the biochemical findings of the study.

### 3.2. mRNA Expression of Apoptosis and Collagen Synthesis-Related Genes

The effects of DLM and two different doses of HSP administered in combination with DLM on mitochondria-dependent apoptosis and mRNA expression of collagen-related genes in testicular tissues were analyzed using RT-qPCR. The expression level of the *Bax* gene was found to be 2.84-fold (*p* < 0.001) higher in the DLM group compared to the control group. However, a 1.43-fold (*p* = 0.029) and 1.99-fold (*p* < 0.001) decrease in *Bax* gene mRNA expression were determined in the DLM + HSP 100 mg/kg and DLM + HSP 300 mg/kg groups, respectively, compared to the DLM group.

When evaluating *Bcl-2* gene mRNA expression in the DLM group compared to the control, a decrease of 3.3-fold (*p* = 0.001) was observed. The 1.97-fold increase (*p* = 0.310) observed in the DLM + HSP 100 mg/kg group compared to the DLM group was not statistically significant. In contrast, the 2.72-fold increase (*p* = 0.006) in the DLM + HSP 300 mg/kg group was statistically significant.

The mRNA expression levels of the *COL1A1* gene were elevated 2.74-fold (*p* < 0.001) in the DLM group compared to the control group. In the DLM + HSP 100 mg/kg and DLM + HSP 300 mg/kg groups, a reduction of 1.65-fold (*p* = 0.011) and 2.06-fold (*p* < 0.001), respectively, was seen compared to the DLM group.

**Figure 2:**
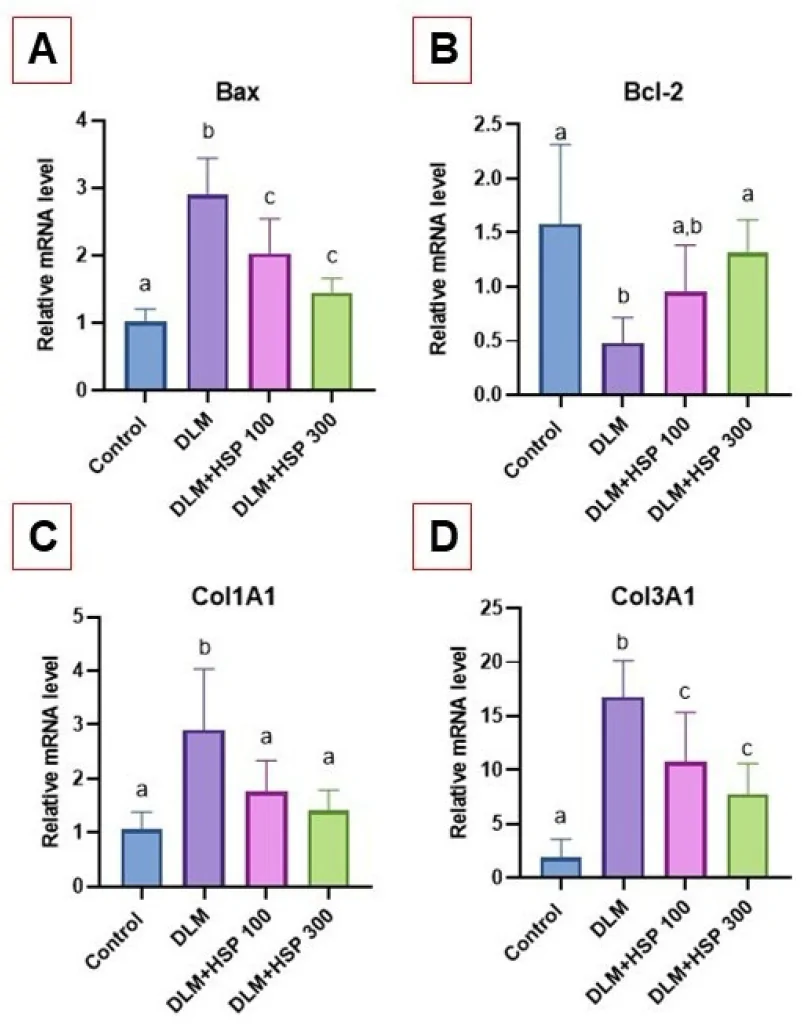
RT-qPCR analysis of the mRNA expression level of (**A**) *Bax*, (**B**) *Bcl-2*, (**C**) *COL1A1*, and (**D**) *COL3A1* among groups (*n* = 8 animals per group). There is a statistically significant difference between the groups not sharing the same letter (*p* < 0.05). Data are presented as mean ± SD. Abbreviations: *Bax*: Bcl-2-associated X protein; *Bcl-2*: B-cell lymphoma 2; *COL1A1*: Collagen type I alpha 1 chain; *COL3A1*: Collagen type III alpha 1 chain; DLM: Deltamethrin; HSP: Hesperidin; mRNA: Messenger RNA; RT-qPCR: Reverse transcription quantitative polymerase chain reaction; and SD: Standard deviation.

The evaluation of *COL3A1* mRNA gene expression revealed a 9.04-fold increase (*p* < 0.001) in the DLM group compared to the control group. In comparison to the DLM group, a reduction of 1.55-fold (*p* = 0.006) and 2.15-fold (*p* < 0.001) was noted in the DLM + HSP 100 mg/kg and DLM + HSP 300 mg/kg groups, respectively. The findings belonging to RT-qPCR are presented in Figure 2.

### 3.3. Histopathological Evaluation

**Figure 3:**
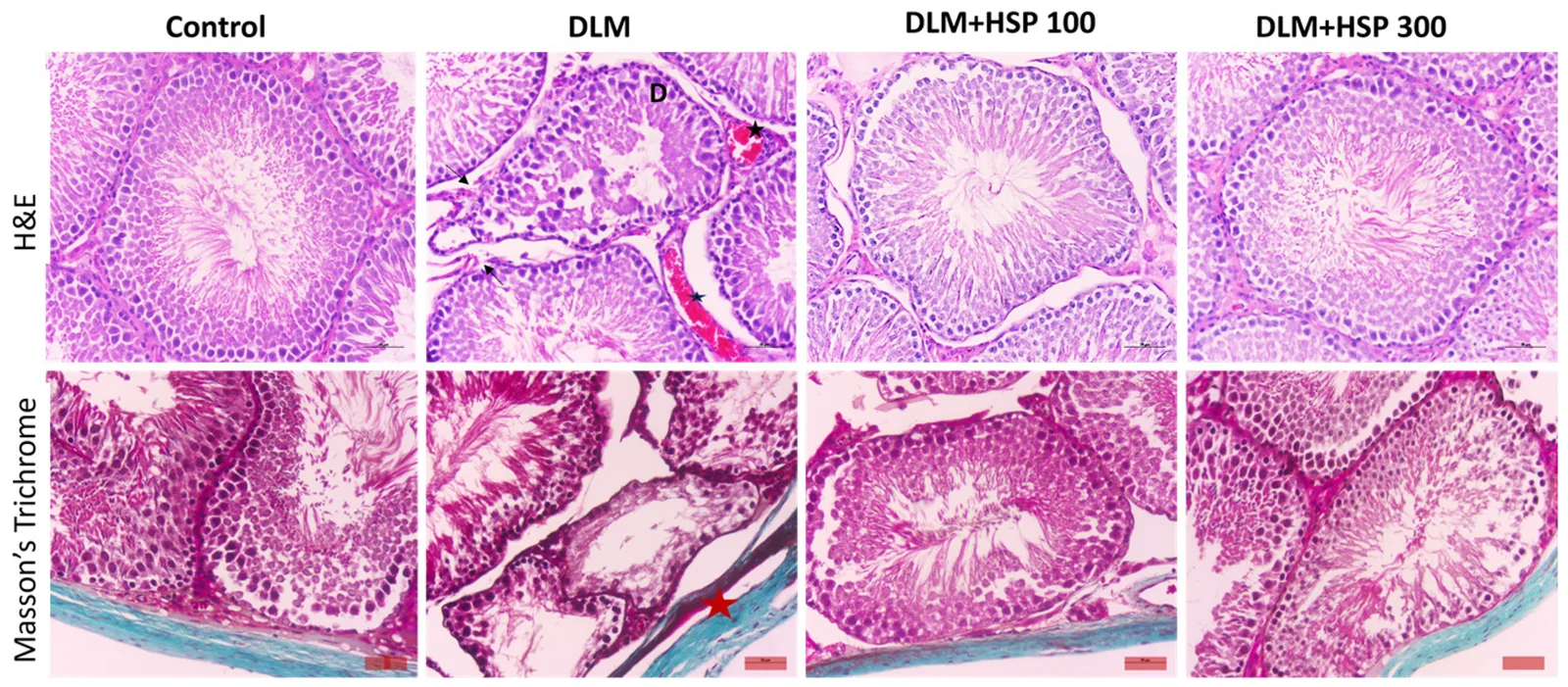
H&E and Masson’s trichrome staining of testicular slides belonging to groups (*n* = 8 animals per group). Representative H&E and Masson’s trichrome sections from the Control, DLM, HSP 100, and HSP 300 groups. The control group shows normal testicular organization with orderly seminiferous tubules, intact germinal epithelium and interstitial compartments, with a continuous basement membrane. In the DLM group, degenerative (D) seminiferous tubules are dysmorphic, and exhibit disrupted germ cell integrity, interstitial congestion (black stars), and disruption of the basement membrane (black arrows). Masson’s trichrome shows fibrotic remodeling in the DLM group, thickened and distorted tunica albuginea depicted with a red star. Scale bars = 50 μm. Abbreviations: DLM: Deltamethrin; H&E: Hematoxylin-eosin; and HSP: Hesperidin.

The control group had normal testicular morphology and structure, including organized seminiferous tubules, intact germinal epithelium and interstitium, normal spermatogenesis, and unchanged basement membrane. Conversely, the DLM group exhibited significant histopathological damage, characterized by tubular disorganization, germ cell detachment/sloughing, vacuolization, interstitial degeneration, and disruption of the basement membrane (Figure 3). In the DLM + HSP 100 and DLM + HSP 300 groups, these modifications were mitigated, although residual vacuolization and mild degenerative changes persisted.

**Figure 4:**
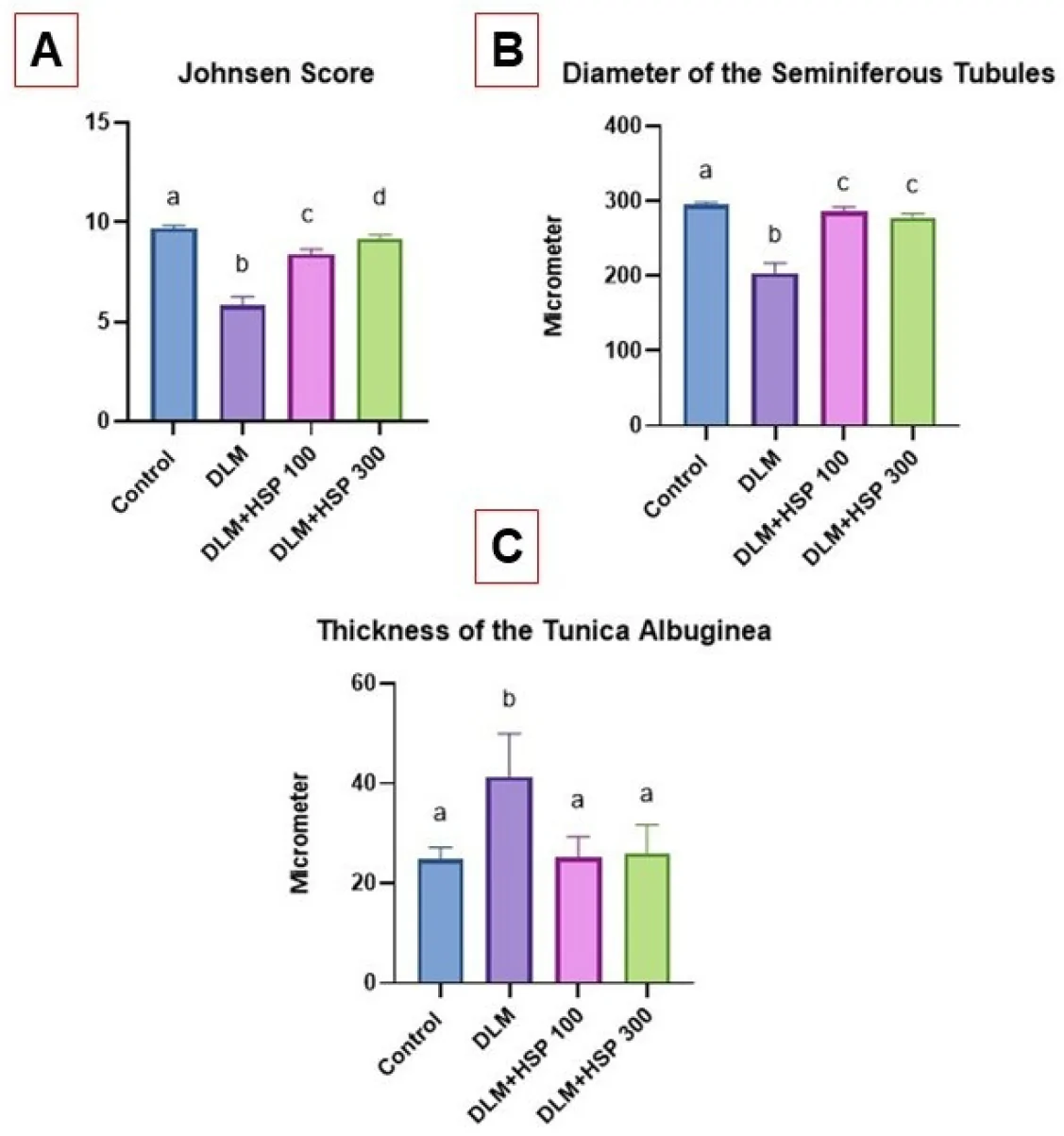
Findings of the histopathological evaluation among experimental groups (*n* = 8 animals per group). (**A**) Johnsen scores of groups; (**B**) Diameter of the seminiferous tubules; and (**C**) Thickness of the tunica albuginea. There is a statistically significant difference between the groups not sharing the same letter (*p* < 0.05). Data are presented as mean ± SD. Abbreviations: DLM: Deltamethrin; HSP: Hesperidin; and SD: Standard deviation.

The mean Johnsen score for the control group was 9.71 ± 0.12, with almost all rats exhibiting complete spermatogenesis. The DLM exposure adversely affected spermatogenesis, yielding a Johnsen score of 5.83 ± 0.45, significantly lower than the control (*p* < 0.001). The mean Johnsen scores for DLM + HSP 100 mg/kg (8.41 ± 0.23) and DLM + HSP 300 mg/kg (9.19 ± 0.18) were higher than those of the DLM group (*p* < 0.001 for both). While HSP effectively mitigated the impairment induced by DLM at both dosages, its effect was notably enhanced at 300 mg/kg in comparison to 100 mg/kg (*p* < 0.001) (Figure 4A).

Histopathological evaluation also revealed that the diameters of the seminiferous tubules were significantly reduced in the DLM group compared to the control group (*p* < 0.001). Nonetheless, HSP treatment at both doses restored this alteration (*p* < 0.001 for both). No significant difference was seen between the two different doses of HSP for seminiferous tubule diameter (*p* > 0.05) (Figure 4B). The thickness of the tunica albuginea in the DLM group was significantly greater than in the control group (*p* = 0.004). DLM + HSP 100 mg/kg and DLM + HSP 300 mg/kg reduced the thickness of the tunica albuginea, which was increased by DLM (*p* = 0.004 and *p* = 0.007, respectively) (Figure 4C).

### 3.4. Immunofluorescence Evaluation

**Figure 5:**
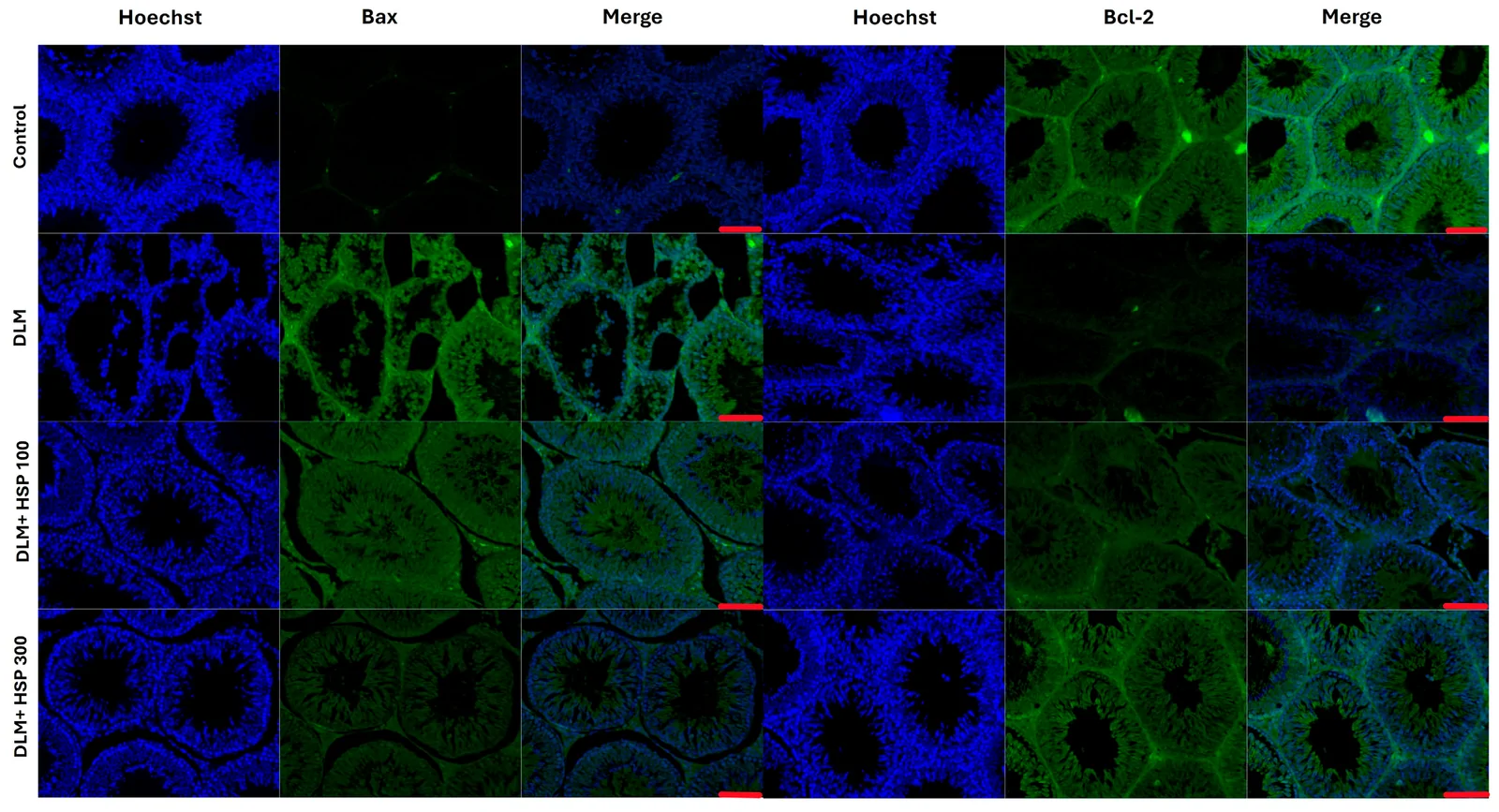
Immunofluorescence staining of Bax and Bcl-2 among groups (*n* = 8 animals per group). Scale bars, 100 μm. Abbreviations: Bax: *Bcl-2*-associated X protein; Bcl-2: B-cell lymphoma 2; DLM: Deltamethrin; and HSP: Hesperidin.

Figure 5 displays immunofluorescence staining images illustrating the expression of Bax and Bcl-2 in the groups. The Hoechst staining of the nuclei appears blue, whilst the antibody staining intensities are depicted in green.

**Figure 6:**
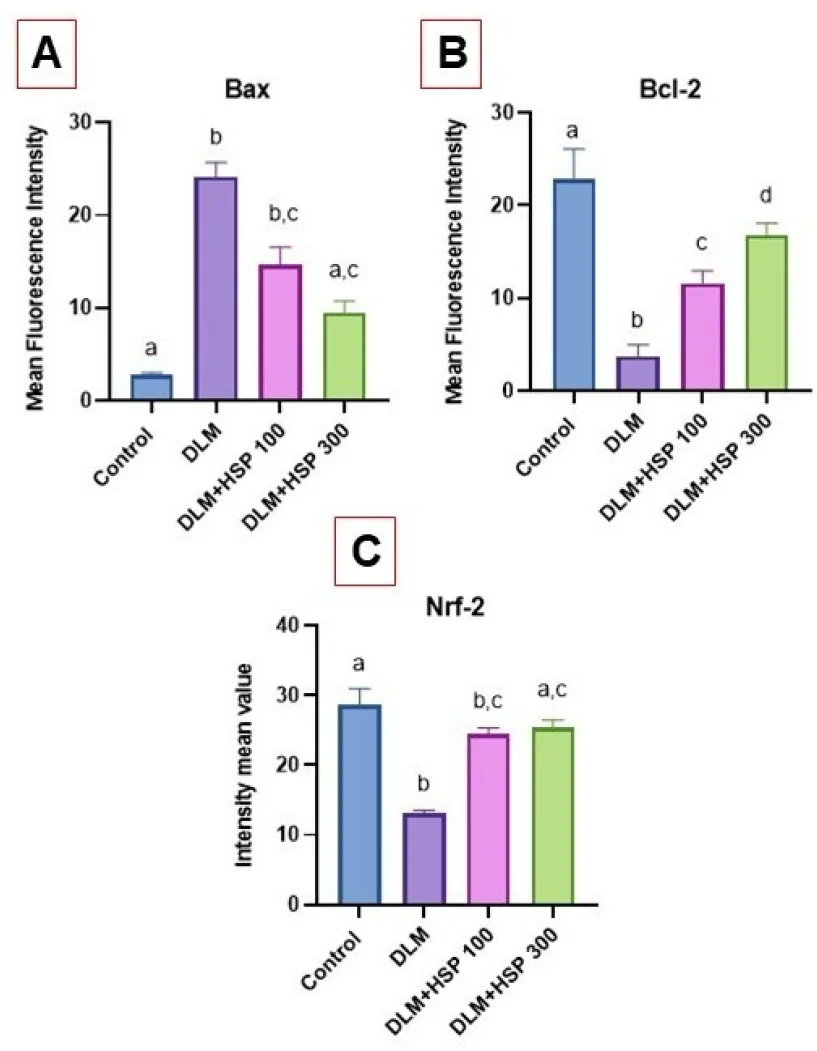
Findings of the IF assessment belonging to experimental groups (*n* = 8 animals per group). Mean intensity values of (**A**) Bax; (**B**) Bcl-2; and (**C**) Nrf-2 expression. There is statistically significant difference between the groups not sharing the same letter (*p* < 0.05). Data are presented as mean ± SD. Abbreviations: Bax: *Bcl-2*-associated X protein; Bcl-2: B-cell lymphoma 2; DLM: Deltamethrin; HSP: Hesperidin; Nrf-2: Nuclear factor erythroid 2–related factor 2; and SD: Standard deviation.

The DLM group exhibited a significantly elevated expression of Bax, a pro-apoptotic factor, compared to the control (*p* < 0.001). While HSP reversed DLM-induced apoptotic changes at a dose of 300 mg/kg, it did not ameliorate apoptosis-related changes at a dose of 100 mg/kg (*p* = 0.004 and *p* > 0.05, respectively) (Figure 6A).

Bcl-2, an anti-apoptotic factor, was seen to be more reduced in the DLM group than in the control group (*p* < 0.001). HSP at both doses had higher levels of Bcl-2 compared to the DLM group (*p* < 0.001 for both) (Figure 6B).

**Figure 7:**
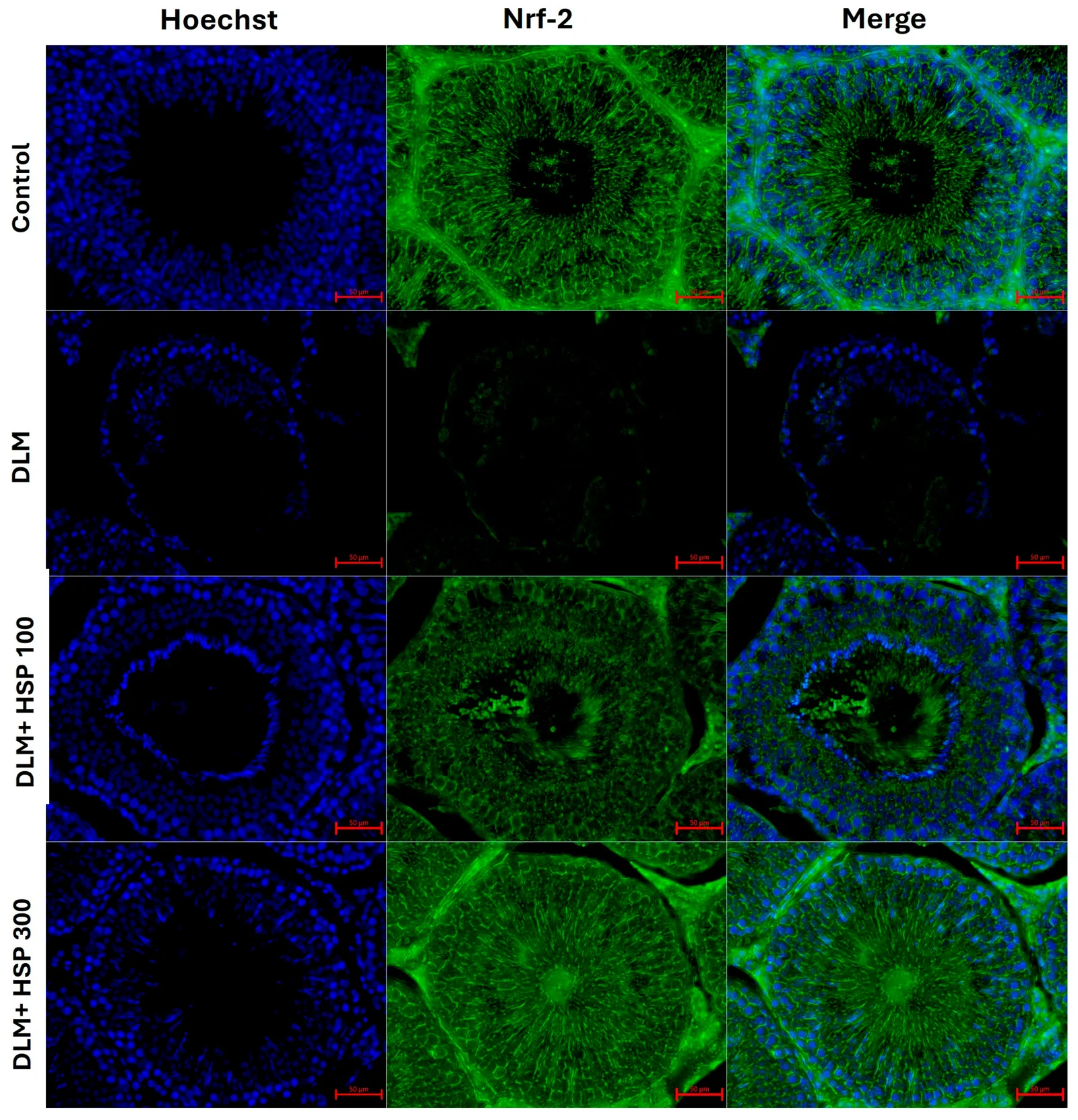
Immunofluorescence staining of Nrf-2 among groups (*n* = 8 animals per group). Scale bars, 50 μm, ×200. Abbreviations: DLM: Deltamethrin; HSP: Hesperidin; and Nrf-2: Nuclear factor erythroid 2–related factor 2.

Figure 7 illustrates the expression of Nrf-2 in the groups, highlighting the relative variations in its expression as assessed using immunofluorescence labeling. The DLM group exhibited reduced Nrf-2 expression compared to the control group (*p* < 0.001). HSP at a dosage of 300 mg/kg significantly elevated Nrf-2 expression compared to the DLM group (*p* = 0.012), although a dosage of 100 mg/kg did not exhibit this effect (*p* > 0.05) (Figure 6C).

## 4. Discussion

The present study demonstrated that DLM induced testicular injury in rats. The observed testicular damage was accompanied by increased systemic oxidative stress markers, alterations in apoptosis-associated signaling, and increased profibrotic gene expression. HSP administration dose-dependently ameliorated these alterations and exerted protective effects against DLM-induced testicular injury. These biochemical and molecular findings were supported by improvements in the histological architecture of the testes and preservation of seminiferous tubule morphology. Overall, the findings suggest that the protective effects of HSP may be associated with modulation of systemic oxidative status, apoptosis-associated signaling, and profibrotic molecular responses.

Oxidative stress has been implicated as one of the major mechanisms contributing to DLM-induced testicular toxicity [21]. In the present study, DLM exposure was associated with increased serum oxidative stress markers, reflecting systemic oxidative status. These findings suggest that systemic oxidative stress may contribute to the observed testicular injury. HSP administration improved serum oxidative stress parameters, indicating a potential role in modulating systemic oxidative status. This effect is primarily associated with a decrease in TOS rather than an increase in TAC, suggesting that the change in redox balance is mainly due to the reduced oxidative load. OSI, which reflects oxidative status in a more general sense, also supports this interpretation. Histological evaluation revealed that Nrf-2 expression was suppressed by DLM administration but increased after HSP treatment. The fact that this effect was observed at a higher dose (300 mg/kg) indicates that the possible involvement of cellular defense mechanisms is dose dependent. The absence of a similar alteration in TAC values may suggest that Nrf-2-mediated responses are tissue-specific or at a regulatory level that cannot be determined by systemic markers.

The cellular Nrf-2/Keap1 pathway is employed to protect against oxidative damage caused by xenobiotics. Elevated levels of ROS can disturb redox equilibrium, resulting in oxidative injury, inflammation, and apoptosis. The activation of Nrf-2 promotes the transcription of Phase II detoxification and antioxidant enzymes, including HO-1 and NQO1. Thus, cells can acclimatize to chemical injury [22]. Consistent with previous reports, DLM exposure in the present study was associated with increased serum oxidative stress markers and reduced Nrf-2 immunoreactivity in testicular tissue [23,24]. Many bioactive compounds have been shown to be effective in correcting DLM-related testicular toxicity due to their antioxidant properties [25,26]. They have achieved this effect by increasing systemic antioxidant reserves or simply by limiting oxidant production, which supports the protective mechanism observed here [27,28]. The concurrent changes in Nrf2 immunoreactivity and systemic oxidative stress markers further support the involvement of oxidative stress-related cellular responses in DLM-induced testicular injury [29]. Taken together, these findings may suggest that the key mechanisms of HSP’s effect in reversing DLM-induced testicular toxicity may be the suppression of oxidant production and dose-dependent modulation of endogenous defense systems. The fact that oxidative stress is a trigger for mitochondrial-mediated apoptosis may explain the efficacy of HSP in modulating apoptotic pathways.

In this study, DLM induced molecular changes associated with apoptosis, resulting in an increase in *Bax* expression and a decrease in *Bcl-2* levels at both the protein and gene expression levels. HSP modulated these changes, with its greater efficacy at a dose of 300 mg/kg, indicating the regulation of apoptosis is in a dose-dependent manner. HSP at a dose of 100 mg/kg had a positive effect on *Bax* levels in terms of gene analysis but had no effect at the tissue level, while the opposite was observed for *Bcl-2*. This suggests that molecular and tissue-level responses may show dose- and context-dependent differences. These findings corroborate previous studies demonstrating that DLM exposure causes an imbalance between *Bax* and *Bcl-2* [5,30]. Antioxidants like rutin and allicin have been shown to inhibit apoptosis in testicular damage by modulating the *Bax/Bcl-2* balance [27]. The current investigation also revealed that HSP has ameliorative effects on the apoptotic alterations induced by DLM in a dose-dependent fashion.

Flavonoid-derived compounds may modulate the equilibrium between Bax and Bcl-2, pro-apoptotic and anti-apoptotic mediators, thus facilitating cell survival under oxidative stress conditions. Furthermore, recent evidence suggests that acetylation-related regulatory mechanisms may influence the modulation of apoptotic signaling pathways linked to oxidative damage. Yang et al. suggested that the protective properties of HSP may involve the regulation of apoptosis-related molecular pathways through this mechanism [31]. Although these mechanisms were not directly investigated in the present study, the findings suggest that the protective effects of HSP may be associated with modulation of systemic oxidative status and Bax/Bcl-2-associated apoptotic signaling.

The increased systemic oxidative stress markers and alterations in apoptosis-associated signaling observed in the present study may contribute to inflammatory responses and profibrotic molecular changes in testicular tissue. In this context, the upregulation of *COL1A1* and *COL3A1*, which are fibrosis-related genes, suggests activation of extracellular matrix accumulation and early fibrotic processes. HSP administration improved these changes at both doses. These molecular improvements were also confirmed by Masson trichrome staining, which showed that HSP administration reduced collagen accumulation and fibrotic changes. Similar to the literature, inflammation and fibrosis play an important role in the pathogenesis of DLM-associated testicular toxicity. The administration of HSP in groups suggests a downregulation of *COL1A1* and *COL3A1*, indicating a possible reduction in collagen deposition and fibrotic remodeling. This may be mechanistically linked to the reduction in the oxidative stress–inflammation–fibrosis axis, as oxidative stress can enhance inflammatory signals and profibrotic extracellular matrix responses. HSP may help to restore the disruption, which is caused by fibrotic changes, due to its anti-fibrotic properties as documented in the literature [32,33].

The histopathological results reveal complementary evidence supporting the biochemical and molecular results of this study. DLM exposure resulted in marked disruption of testicular architecture, as demonstrated by reduced seminiferous tubule diameter and increased thickness of the tunica albuginea. It also decreased Johnsen scores, which indicate impaired spermatogenesis. In contrast, HSP treatment ameliorated all these disruptions. The consistency between all findings, including molecular, biochemical, and histological strengthens the fact that HSP exerts a versatile protective effect on testicular tissue. The present study indicates that the preservation of testicular structure is closely linked to the modulation of interconnected oxidative, apoptotic, and fibrotic pathways.

This study has several limitations. Firstly, the number of experimental animals and groups was limited due to institutional resources. In this regard, it was not possible to form groups such as a group with only HSP administered and a group with no intervention/administration, including corn oil. Secondly, although biochemical evaluation of serum samples, particularly of oxidative stress parameters, allows for systemic assessment, it has proven insufficient for determining local tissue redox status. Therefore, our findings regarding oxidative stress should be interpreted with caution. Future studies including tissue-specific oxidative stress markers such as MDA, GSH/GSSG ratio, SOD, CAT, and GPx activities would provide stronger mechanistic evidence. Additionally, although increased Nrf-2 immunofluorescence was observed, downstream *Nrf-2* target genes (e.g., HO-1 and NQO1) and nuclear translocation were not evaluated. Therefore, activation of the Nrf-2 signaling pathway could not be directly confirmed, and the involvement of Nrf-2 should be interpreted as a potential mechanism rather than definitive evidence. Moreover, it should be noted that while CRP levels were measured in this study, CRP is considered a minor acute-phase reactant in rodents compared to humans. Furthermore, clinical analyzer kits validated for human samples may have lower analytical sensitivity for rodent CRP. Therefore, these CRP findings should be interpreted with caution as exploratory observations, and future studies utilizing rodent-specific cytokine panels (e.g., IL-6, TNF-alpha) are recommended to validate these responses. Finally, the absence of spermatogenic parameters, including sperm count, motility, morphology, and fertility-related outcomes constitutes an additional limitation. In addition to the lack of spermatological evaluation, testicular weight and reproductive hormone levels (e.g., testosterone, LH, and FSH) were not assessed. These parameters would provide complementary functional evidence regarding testicular injury and recovery. Further research is necessary to elucidate the long-term impacts of HSP and to more precisely delineate the molecular mechanisms underlying its protective function. Moreover, future experimental and clinical investigations could clarify its potential therapeutic efficacy and relevance in the prevention of testicular damage.

## 5. Conclusions

This study demonstrated that HSP may alleviate DLM-induced testicular damage in a dose-dependent manner. These findings expand the current understanding of the potential protective effects of HSP against DLM-induced testicular injury by providing an integrated evaluation of systemic oxidative stress, apoptosis-associated signaling, profibrotic molecular changes, and histopathological alterations. The present findings may serve as a foundation for future experimental studies aimed at elucidating the underlying molecular mechanisms and evaluating the translational potential of HSP in toxicant-induced male reproductive injury.

## Figures and Tables

**Table 1:** Primers nucleotide sequences of genes used in RT-qPCR analysis.

Gene	Forward Primer (5′-3′)	Reverse Primer (5′-3′)
*Bax*	GATGGCCTCCTTTCCTACTTC	CTTCTTCCAGATGGTGAGTGAG
*Bcl-2*	GGAGGATTGTGGCCTTCTTT	GTCATCCACAGAGCGATGTT
*COL1A1*	CCAATGGTGCTCCTGGTATT	GTTCACCACTGTTGCCTTTG
*COL3A1*	GTGTGATGATGAGCCACTAGAC	TGACAGGAGCAGGTGTAGAA
*GAPDH*	GCATTGCAGAGGATGGTAGAG	GCGGGAGAAGAAAGTCATGATTAG

## Data Availability

All data generated or analyzed during this study are included in this article. Further inquiries can be directed to the corresponding author.

## Abbreviations

The following abbreviations are used in this manuscript:

DLM	Deltamethrin
HSP	Hesperidin
Nrf-2	Nuclear factor erythroid 2–related factor 2
ROS	Reactive oxygen species
RT-qPCR	Real-time quantitative PCR
TAC	Total antioxidant capacity
TOS	Total oxidant status
OSI	Oxidative stress index
CRP	C-reactive protein
H&E	Hematoxylin-eosin
Bax	Bcl-2-associated X protein
Bcl-2	B-cell lymphoma 2
